# Changes in the Transcriptome and Long Non-Coding RNAs but Not the Methylome Occur in Human Cells Exposed to *Borrelia burgdorferi*

**DOI:** 10.3390/genes15081010

**Published:** 2024-08-01

**Authors:** Anne Berthold, Vett K. Lloyd

**Affiliations:** Department of Biology, Mount Allison University, Sackville, NB E4L 1G7, Canada; aberthold@mta.ca

**Keywords:** transcriptome, epigenetics, Lyme disease, *Borrelia burgdorferi*, HUVECs, HEK-293 cells, lncRNAs

## Abstract

Lyme disease, caused by infection with members of the Lyme borreliosis group of *Borrelia* spirochete bacteria, is increasing in frequency and distribution worldwide. Epigenetic interactions between the mammalian host, tick, and bacterial pathogen are poorly understood. In this study, high-throughput next-generation sequencing (NGS) allowed for the in vitro study of the transcriptome, non-coding RNAs, and methylome in human host cells in response to *Borrelia burgdorferi* infection. We tested the effect of the *Borrelia burgdorferi* strain B31 on a human primary cell line (HUVEC) and an immortalized cell line (HEK-293) for 72 h, a long-duration time that might allow for epigenetic responses in the exposed human host cells. Differential gene expression was detected in both cell models in response to *B. burgdorferi*. More differentially expressed genes were found in HUVECs compared to HEK-293 cells. *Borrelia burgdorferi* exposure significantly induced genes in the interferon, in addition to cytokine and other immune response signaling in HUVECs. In HEK-293 cells, pre-NOTCH processing in Golgi was significantly downregulated in *Borrelia*-exposed cells. Other significantly altered gene expressions were found in genes involved in the extracellular matrix. No significant global methylation changes were detected in HUVECs or HEK-293 cells exposed to *B. burgdorferi*; however, two long non-coding RNAs and a pseudogene were deregulated in response to *B. burgdorferi* in HUVECs, suggesting that other epigenetic mechanisms may be initiated by infection.

## 1. Introduction

Zoonotic diseases are the focus of a global research effort, in part due to the COVID-19 pandemic and fears of future pandemics. Ticks are efficient vectors for transferring zoonotic pathogens to humans and other animals. One tick-borne disease is Lyme disease or Lyme borreliosis, which is caused by the spirochete *Borrelia burgdorferi* and related species [1]. As ticks spread across temperate regions of the globe including North America and Europe, encounters between humans and ticks are expected to increase, with Lyme disease and other tick-borne diseases becoming an emerging public health concern [2]. The incidence of Lyme disease has increased in recent decades, as evidenced by surveillance data from several European countries, the United States [3,4,5,6,7,8,9,10,11], and the World Health Organization (WHO) office in Europe [12]. The annual number of Lyme disease cases is estimated to be ~65,000 to 85,000 in Europe [12,13] and ~300,000 in the USA [6,7]. Even so, tick-borne diseases are likely under-reported, as access to testing varies within and between countries, the sensitivity and specificity of the most common serological tests are imperfect and geographically variable, and case definitions vary among countries [14,15,16].

Infection with *Borrelia* is divided into three stages, namely early localized, early disseminated, and late disseminated infection [17]. Transmission of tick-borne pathogens occurs primarily by injection as the tick feeds and tick salivary proteins modulate host defense mechanisms [18]. Studies of *B. burgdorferi* have shown that, depending on the cell or animal model, different signaling molecules in the immune response cascade are downregulated, promoting pathogen survival and spread in the host [17,19]. Both local and disseminated multisystemic symptoms place a burden on patients, which is exacerbated in cases where there is lack of risk recognition and accurate diagnosis and treatment of the disease [20]. The symptomatology of the disease is manifold and varies from patient to patient [21]. Even after treatment, ongoing symptoms due to failure to clear the infection, cellular damage/alteration, or altered immune response can occur [22].

Epigenetic processes are central to the regulation of biological processes of an organism, and their misregulation contributes to the development of diseases; the impact on immune function is no exception [23]. The environment is the classic trigger of epigenetic changes, yet the role of ticks in inducing epigenetic changes in their host remains largely unexplored. An organism may be exposed to various environmental factors in the short or long term, including arthropod vectors of bacterial pathogens [24]. These environmental factors leave epigenetic marks that complement the genetic code of the host to determine the level of gene expression and function [25]. Epigenetic mechanisms are complex, highly dynamic, and reversible yet can be transmitted to the next generation [26]. Major epigenetic mechanisms include chemical modifications of histones or of DNA bases, the insertion of histone variants and remodeling complexes, and the action of non-coding RNAs (ncRNAs) [25].

A cell type-specific spatial and temporal resolution of genome functions, including all epigenetic mechanisms and their interplay, is essential for a detailed understanding of normal and pathophysiological states [27]. Bacteria have the ability to alter the epigenome of the host they infect, reprogramming host genes and leading to alterations in host physiology, morphology, and behavior [28]. These adaptations have been selected to be beneficial to bacteria, allowing the bacteria to infect, then persist and spread within the host. For example, Anaplasma bacterial pathogens manipulate physiological processes in their tick vectors by altering histone-modifying enzymes [29]. In astrocytes infected with *B. burgdorferi*, transcriptomic and epigenomic analyses showed that access to chromatin decreased during the course of infection, which was associated with anatomical and morphological changes and, eventually, stress mechanisms in the cells [30]. Thus, epigenetic mechanisms are likely to play an important role in *Borrelia* infection during host–pathogen interactions [28,31].

In nature, ticks contain diverse microbiota, and they can transmit multiple pathogens; the frequency with which this happens varies over time and by geographical location in response to pathogen loads in reservoir species [32]. Opportunistic infections can also occur in response to immune suppression from tick saliva and the resulting disease [33,34,35]. Pathogens can interact with the vector and the host, as well as with each other, in a battle to establish infection and find their niche to survive and propagate [33,34,35]. To simplify some of these complex interactions, studies on *Borrelia* and their hosts have often used cell models. Numerous mammalian cell types have been studied in vitro and in vivo. Studied cells include keratinocytes [36], fibroblasts [37,38,39,40,41], breast cancer cells [42], neuronal cells [43,44,45,46,47,48], and blood-derived cells [49,50,51,52,53,54]. This study focuses on in vitro interactions between *B. burgdorferi* and human cells. We hypothesize that exposure of human cells to bacteria leads to a change in transcription that might be due to altered DNA methylation or altered expression of non-coding RNAs. We used two cell models—one endothelial and one epithelial, as these cell types are expected to respond differently. The epidermis and dermis, as well as the various cell populations therein, represent the first physical barriers that ticks and pathogens must overcome to establish an infection [55]. Human umbilical vein endothelial cells (HUVECs) have previously served as a primary cell model in several pathogen–host interaction studies [56,57] to explore the localization of *Borrelia* in cells [41,47,58] and to study transcriptional changes [59]. Human embryonic kidney cells (HEK-293) have been used to study the attachment of *B. burgdorferi* to extracellular matrix components such as negatively charged polysaccharide glycosaminoglycans (GAGs) and proteoglycans [43,46,60]. We chose an exposure time of 72 h, which is a more prolonged exposure than most other studies with exposure times restricted to between 2 and 24 h [36,38,40,41,44,45,47,50,53], to maximize the host cell’s opportunity to respond epigenetically. We used live bacterial cells because it has been shown that in peripheral blood mononuclear cells, live *B. burgdorferi* 297 induce stronger inflammatory signal than bacterial lysate [53]. By studying both transcriptional and methylome changes in two different human cell types exposed to *B. burgdorferi* for 72 h, we expand knowledge of the cellular interface between the host and tick-borne pathogens.

## 2. Materials and Methods

### 2.1. Human Cell Culture

HUVECs and HEK-293 cells were both originally purchased from the American Type Culture Collection (ATCC) and kindly provided by Gilles Robichaud (Université de Moncton, Moncton, NB, Canada). HUVECs (ATCC, PCS-100-010) required EGM^TM^ BulletKit^TM^ optimized endothelial cell growth media (Lonza, Walkerville, MD, USA, CC-3124) containing EBM^TM^ Basal Medium complemented with 2% *v*/*v* FBS, 0.4% *v*/*v* bovine brain extract and 0.1% *v*/*v* each of hydrocortisone, human epidermal growth factor, ascorbic acid, and gentamicin sulfate amphotericin-B (GA-1000). HEK-293 cells (ATCC, CRL-1573) were grown in DMEM media (Lonza, 12-733F) supplemented with 10% *v*/*v* heat-inactivated FBS (Rockland, Limerick, PA, USA, FBS.02-0500), and 1% *v*/*v* each of L-glutamine (Lonza, Walkersville, MD, USA, 17-605E), sodium pyruvate (Lonza, Walkersville, MD, USA, 13-115E), and penicillin/streptomycin (Sigma-Aldrich, P4333). In our study, human and bacterial cell culture required synchronization. Cells were initially seeded into 75 cm^2^ flasks (Celltreat, Pepperell, MA, USA, 229341) and maintained until 80% confluency with regular media changes every second day before they were counted with trypan blue staining to distinguish living and dead cells (Thermo Fisher Scientific, Grand Island, NY, USA, 15250-061) and seeded into well plates. Cell growth was monitored with an inverted microscope (Motic, Richmond, BC, Canada, AE31). An aliquot of both cell lines was subjected to mycoplasma testing (e-Myco™ VALiD Mycoplasma PCR Detection Kit, Froggabio, Concord, ON, Canada, 25239) and confirmed negative.

### 2.2. Borrelia burgdorferi Culture

*Borrelia burgdorferi* strain B31 (B31; [61,62]) obtained from the American Type Culture Collection (ATCC, 35210) was grown in optimized nutrient-rich Barbour–Stoenner–Kelly medium including 6% rabbit serum (BSK-H, Darlynn Biologicals, BB83-500) supplemented with antibiotics (Sigma-Aldrich, Saint Louis, MO, USA ) to a final concentration of 50 µg/mL rifampicin (R3501), 20 µg/mL phosphomycin (P5396), and 2.5 µg/mL amphotericin B (A9528) in glass culture tubes, as described in [63]. Growth and motility were monitored with a Leitz phase-contrast microscope, and bacteria were counted with a hemacytometer at 250× magnification. All exposure experiments were performed with *B. burgdorferi* strain B31 in an early passage (4 or 5 from original culture) in their exponential growing phase at a concentration of 2 to 4 × 10^7^ cells/mL, grown in liquid medium at 34 °C without shaking, aeration, or CO_2_.

### 2.3. Human Cell Exposure Experiment

**Figure 1:**
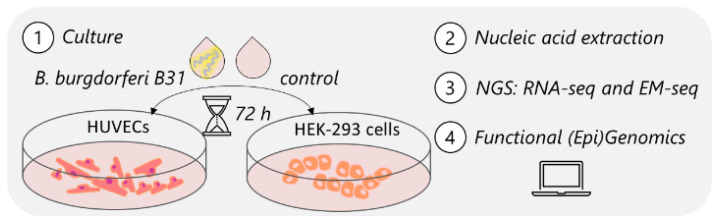
Experimental workflow for human cell models exposed to *B. burgdorferi* B31. HUVECs and HEK-293 cells were exposed to *B. burgdorferi* B31 for 72 h. RNA and DNA were extracted and subjected to library preparation for next-generation sequencing (RNA-seq and enzymatic methyl-seq). Genome-wide epigenomic and transcriptomic data were used for functional enrichment analysis.

As shown in Figure 1, starting materials for the exposure experiments were HUVECs in passage 3 and HEK-293 cells in passage 5 seeded at a density of 4000 cells/cm^2^ into 6-well tissue culture plates (Celltreat, 229105) in antibiotic-containing media. The three independent replicates of cell exposure experiments were performed with cells of the same passage. At a density of 1.2 × 10^5^, human cells were exposed to *B. burgdorferi* strain B31 with a multiplicity of infection (MOI) of bacteria cells to human cells of 50:1. To the best of our knowledge, there is also no quantitatively validated study on how many *Borrelia* infect human cells. Presumably, not all of tick-borne pathogens transfer from the tick to the host. A 2020 study quantified *Borrelia* spirochetes using the LUX real-time PCR assay and found between 2.0 × 10^0^ and 7.0 × 10^5^ *Borrelia* cells per tick collected from migratory birds [64]. Even if all bacteria cells are transmitted, once in the host, there are multiple host cells and a complex extra-cellular environment with which the *Borrelia* interact. The *Borrelia* dose per cell, defined as the MOI, used in different studies ranges from 1 to 1000 [36,38,40,41,53,65,66,67]. Consistent with previous studies, we found that human cells were not apoptotic at MOIs of 300 or 600 bacteria per cell at 24 and 48 h.

The adherent mammalian cells were washed with PBS (Thermo Fisher Scientific, 14190144) before the addition of resuspended bacteria. *Borrelia burgdorferi* strain B31 cultures were counted and centrifuged at 4500× *g* for 10 min, and 6 × 10^6^ bacteria were used per well. The pelleted bacteria were resuspended in human cell growth medium (EBM for HUVECs, DMEM for HEK-293 cells) without antibiotics (gentamicin-amphotericin or penicillin/streptomycin). In parallel, the cells were incubated with growth media only (without antibiotics) as a control. All plates were incubated for 72 h under constant environmental conditions of 37 °C, 5% CO_2_, and humidified air. Human cell growth and morphology were monitored microscopically. After the 72 h exposure, the wells of bacterially treated or control cells for each cell type were washed with PBS and trypsinized, and pairs of wells from the same treatment were combined in a 15 mL reaction tube to yield approximately 2 × 10^6^ cells for nucleic acid extraction. Cells were pelleted by centrifugation at 300× *g* for 5 min, supernatant was removed, and cell pellets were kept at −80 °C until further use.

### 2.4. Nucleic Acid Extraction and Assessment

RNA extraction was performed on each replicate with the RNeasy Mini Kit (74104, Qiagen, Hilden, Germany), following the manufacturer’s instructions, including DNase I on-column digestion (1010395, Qiagen) and elution in 50 µL RNase-free water. The quality and quantity of RNA were initially assessed with a Nanodrop 1000 (Thermo Fisher Scientific) and denaturing RNA-gel. RNA quality was additionally assessed for integrity and size distribution using the Tapestation RNA assay (Agilent, Santa Clara, CA, USA, 5067-5576 and 5067-5577), while RNA concentration was determined using the RNA HS assay from Qubit (Thermo Fisher, Life Technologies, Q32852). Samples with RIN ≥ 9 were used for library preparation.

DNA extraction was performed with a DNeasy Blood and Tissue Kit (69506, Qiagen), following the manufacturer’s instructions, including an RNase A treatment (R6513, Sigma-Aldrich) and extraction in 100 µL of Tris-HCl (pH 8) with 0.1 mM EDTA. DNA concentration was initially estimated spectrophotometrically with a Nanodrop 1000. Next, the quality of the DNA was assessed using a Tapestation Genomic DNA assay (Agilent, 5067-5365 and 5067-5366) for integrity and size distribution. Concentration was determined with a dsDNA BR assay from Qubit (Thermo Fisher, Life Technologies, Q32850). Samples with DIN ≥ 8 were used for library preparation.

### 2.5. Library Preparation

#### 2.5.1. RNA-Seq

The library for RNA-seq was prepared with 200 ng RNA input and an NEBNext Ultra II Directional RNA Library Prep Kit for Illumina (New England BioLabs (NEB), Ipswich, MA, USA, E7760L) at the Atlantic Cancer Research Institute, Moncton, New Brunswick, Canada. First, mRNA was enriched using the NEBNext Poly (A) mRNA Magnetic Isolation Module (NEB, E7490L), following the manufacturer’s instructions. Briefly, after isolation, samples were fragmented for 15 min at 94 °C, targeting an insert size of 200 bp. Next, the first and second cDNA strands were synthetized, followed by clean-up with Ampure XP beads (Beckman Coulter, Brea, CA, USA, A63881). Then, the samples were end-prepped, and the adaptor was ligated. The adaptor was diluted following the manufacturer’s instructions. The adaptor-ligated samples were purified with Ampure XP beads before amplification. A different Unique Dual Index Primer Pair (NEB, E6440S) was added to each sample, and 11 cycles were used for PCR enrichment. Purification using Ampure XP beads was used to obtain the final library. The quality and quantity of the library were assessed with a Tapestation D1000 assay (Agilent, 5067-5582 and 5067-5583) and a dsDNA HS assay from Qubit (Thermo Fisher, Life Technologies, Q32851).

#### 2.5.2. Enzymatic Methyl-Seq (EM-Seq)

The library for EM-seq was prepared using 200 ng DNA input and an NEBNext Enzymatic Methyl-seq Kit (NEB, E7120S), following manufacturer’s instructions for a standard insert (370–420 bp). First, a CpG-methylated pUC19 control and unmethylated lambda control were diluted 100 times in TE Buffer (pH 8.0) (Thermo Fisher, Life Technologies, 12090015) to final concentrations of 0.001 ng/µL and 0.02 ng/µL, respectively. Then, DNA samples and diluted pUC19 and lambda controls were fragmented using a *Covaris E210* Focused Ultrasonicator (Covaris) for a target insert size of 350 bp. After shearing, 1 µL each of pUC19 and lambda controls were added to each DNA sample. Samples were end-repaired and dA-tailed, and the adaptor was ligated. The adaptor-ligated samples were purified with NEBNext Sample Purification Beads. An EM-seq conversion reaction was performed following manufacturer’s instructions, starting with oxidation of 5-methylcytosines and 5-hydroxymethylcytosines. DNA-converted samples were purified before denaturation. For denaturation, sodium hydroxide was used, immediately followed by the deamination of cytosines. The deaminated samples were purified before being amplified. For the PCR enrichment, a different EM-Seq Index Primer (NEB, E7120S) was added to each sample, and 4 cycles were used. A final purification using NEBNext Sample Purification Beads was performed to obtain the final library. Size distributions of libraries were determined with a Tapestation D1000 assay (Agilent, 5067-5582 and 5067-5583), and the library concentration was evaluated with a dsDNA HS assay from Qubit (Thermo Fisher, Life Technologies, Q32851). Both library preparation and the quality check were performed at the Atlantic Cancer Research Institute, Moncton, New Brunswick, Canada.

### 2.6. Sequencing

#### 2.6.1. RNA-Seq

Whole-genome RNA-seq and EM-seq were performed at the Next Generation Sequencing Core Facility at the Atlantic Cancer Research Institute, Moncton, New Brunswick, Canada. Equimolar amounts of libraries for RNA-seq were first sequenced on an iSeq 100 instrument (Illumina, San Diego, CA, USA) using 2 × 80 paired-end sequencing to assess both library and pooling qualities. Library inputs were rebalanced following Illumina’s recommendations to ensure equal representation of each sample. Libraries were then sequenced using a NovaSeq 6000 instrument (Illumina). Samples were loaded on an SP flow cell, and 2 × 80 paired-end sequencing was used. The XP workflow was utilized, and samples were distributed as 12 samples on one lane for approximately 25M paired-end reads/sample.

#### 2.6.2. EM-Seq

Equimolar amounts of libraries for enzymatic methyl-seq were first sequenced on an iSeq 100 instrument (Illumina) using 2 × 151 paired-end sequencing to assess both library and pooling qualities. Library inputs were rebalanced following Illumina’s recommendations to ensure equal representation of each sample. Libraries were then sequenced using a NovaSeq 6000 instrument (Illumina). Samples were loaded on an S2 flow cell, and 2 × 151 paired-end sequencing was used.

### 2.7. Data Analysis

#### 2.7.1. RNA-Seq

The quality of the raw reads was verified with FastQC (v0.11.9). Next, adapter trimming with BaseSpace™ Sequence Hub Prep platform was followed by trimming with Trim Galore (v.0.4.4, [68]) with default settings. Trimmed reads were used as input data in Salmon (v.1.4.0, [69]) to perform pseudo alignment to the human transcriptome from Gencode (Release 37 - GRCh38.p13) and quantification. The quantification data were uploaded in the R statistical environment (v.4.0.3, [70]) using Bioconductor package Tximeta (v.1.10, [71]). Low-expression genes were filtered, and only genes with at least more than 1 read in at least 2 samples in one of the groups were considered for further analysis. The normalization and differential expression of the count matrix (an equal number of sequence fragments assigned to each gene]) was performed with DESeq2 (v.1.28.1, [72]). We considered genes with p_adj_(FDR) < 0.05 and with log2FC ≥ |1| as differentially expressed (DE). The gplots (v.3.1.1, [73]) and ggplot2 (v.3.3.3, [74]) packages were used to generate both the heat map and the volcano plot. The Reactome database (v.1.7.0, [75]) was used to identify differentially expressed pathways in humans with a t-test and p-value adjustment according to the Benjamini–Hochberg formula. The ClueGO App (v.2.5.8 [76]) in Cytoscape (v.3.8.2 [77]) was used to visualize functionally grouped networks of signaling pathways relying on Reactome input. For the comparative transcriptome study, the online tool available at https://bioinformatics.psb.ugent.be/cgi-bin/liste/Venn/calculate_venn.htpl was used (accessed 8 July 2022).

#### 2.7.2. EM-Seq

Fastq sequence files were generated in Illumina BaseSpace. Reads were aligned (directionally) to the human (hg19 UCSC ALT-AWARE), pUC19, and lambda genomes using the Dragen methylation pipeline (methyl-seq, v.3.7.5) in Illumina BaseSpace using the default Bismark parameters for methylation reporting. Alignments for spike-in (pUC19 and lambda) genomes were performed determine the conversion efficiency with an NEB EM-seq kit. Following the alignment, the data were further analyzed using the MethylKit (v.2.0.2, [78]) app in Illumina BaseSpace with a minimum CpG coverage of 5× or 10×, testing the 10% to 25% methylation difference and a *q*-value of 0.01 or 0.05.

## 3. Results

### 3.1. Human Cells Exposed to B. burgdorferi Strain B31 Show Altered Gene Expression

HUVECs and HEK-293 cells are widely used human cell models. As illustrated in Figure 1, we exposed HUVECs and HEK-293 cells to *B. burgdorferi* strain B31 for 72 h to monitor and assess the response, specifically targeting changes in gene expression and concomitant DNA methylation changes.

**Figure 2:**
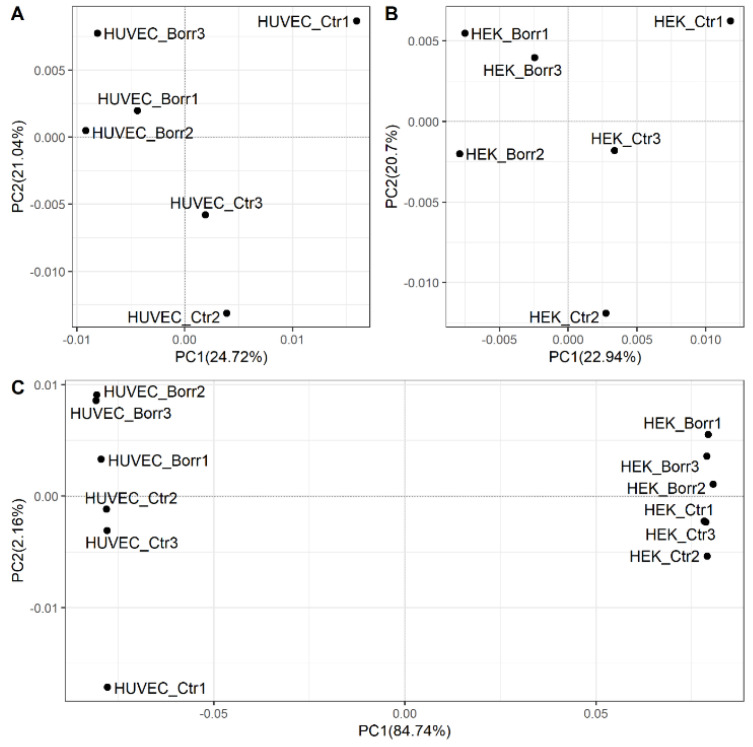
PCA of transcriptome data. Human HUVEC and HEK-293 human cell models remained unexposed (control; Ctr) or were exposed to *B. burgdorferi* strain B31 for 72 h (Borr). Experiments were performed in triplicate, as indicated by the sample names. Unexposed (Ctr) and *B. burgdorferi*-exposed (Borr) cells (**A**: HUVECs; **B**: HEK-293 cells) clustered separately along the x-axis with PC 1, explaining the variation between exposure conditions, while PC2 explained the clustering between replicates in each condition. The positive correlated samples are closer together and are located on the same side of the plot. Comparing HUVECs and HEK-293 cells (**C**), the replicates clustered separately along the x-axis, with PC1 explaining 84.74% of the variation between the cell types. PC2 explained 2.16% of sample correlation clustering. The positively correlated clusters under the HEK-293 cell condition are less extended than the HUVEC clusters.

The RNA-seq study generated 1.07 billion reads (average of 89.57 million reads per sample). Mapping efficiency to the reference genome averaged 97.9%, as documented in Supplemental File S1. Principal component analysis (PCA) was applied to explore the comprehensive expression data and map the correlation in a quantitative 2D graph, after filtering out low-expression gene loci (Figure 2). We first compared the distribution of unexposed and exposed experimental replicates in each cell model individually. We then also included the two cell types and all treatment conditions in the comparison. For each cell type, there was a clustering along principal component (PC) 1, which explains 24.72 and 22.94% of the variation among unexposed control cells and *B. burgdorferi*-exposed HUVECs and HEK-293 cells, respectively (Figure 2A,B). This clustering can be attributed to exposure conditions, which explain the largest variation in the data set. The variation for PC2 could be attributed to the experimental replicates. When HUVECs were compared to HEK-293 cells, including all exposure conditions, 84.74% and 2.16% of the differences could be attributed to cell-type characteristics along PC1 and PC2, respectively (Figure 2C). Thus, the response differs by cell type.

Since the variation difference between cell types was so clear, subsequent analysis treated each cell type separately. Differential expression analysis with DeSeq2 identified the significant changes of the two cell models, HUVECs and HEK-293 cells, in response to *B. burgdorferi* strain B31. The expression data were examined with Relative Log Expression (RLE) plots as a quality control measure to confirm the normalization across samples (Figure S1).

**Figure 3:**
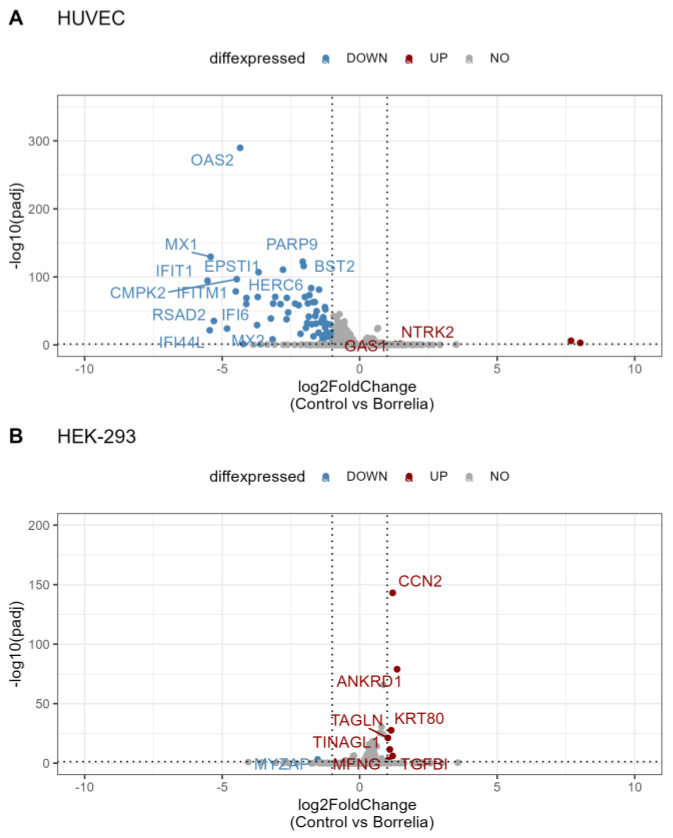
Volcano plot of differential gene expression. Comparison of expression data from HUVECs (**A**) and HEK-293 cells (**B**) unexposed or exposed to *B. burgdorferi* strain B31 for 72 h. log2FC is plotted on the x-axis, representing the level of change between the two exposure conditions, against statistical significance in terms of p_adj_ on the y-axis. The horizontal line marks p_adj_ = 0.05, whereas the vertical lines represent the cut-off of log2FC at -1 and 1. The blue dots represent the genes expressed at a significantly lower level in control cells and upregulated in *Borrelia*-exposed cells, while the red dots represent the genes expressed at significantly higher levels in the control cells and downregulated in the *Borrelia*-exposed cells. The many genes highlighted in gray are outside the cut-offs. HUVECs have considerably more differentially expressed (DE) genes, most of which are expressed at lower levels in control cells and upregulated in response to *Borrelia* exposure. In contrast, in HEK-293 cells, there are more DE genes expressed at higher rather than lower levels in control cells and downregulated in response to *Borrelia*.

A total of 26,919 genes in HUVEC were filtered with *p*-values adjusted for multiple testing using the Benjamini–Hochberg method; *p*_adj_ < 0.05, and 364 differentially expressed (DE) genes were found. Many of these genes were up- or downregulated by less than two-fold, falling in the range of log2 fold change (log2FC) ≤ |1|, but 69 genes had log2FC ≥ |1|. In HEK-293 cells, a total of 29,367 genes were filtered for *p*_adj_ < 0.05, resulting in 110 DE genes, of which 8 had log2FC > |1|. Volcano plots (Figure 3) support the findings of the differential expression analysis and demonstrate that in HUVECs, there are more genes expressed at lower levels in unexposed controls compared to *B. burgdorferi*-exposed cells, meaning that *Borrelia* exposure results in gene upregulation. In contrast, in HEK-293 cells, the majority of significant genes are expressed at higher levels in unexposed control cells compared to *B. burgdorferi*-exposed cells, indicating downregulation with *Borrelia* exposure. Heat maps facilitate visualization of the log2 scaled, mean-centered expression of each of the identified DE genes for each sample replicate in HUVECs (Figure S2) and HEK-293 cells (Figure S3). The heat maps also clearly show that *B. burgdorferi* exposure produces distinct patterns of gene expression, which is supported by the column dendrograms in the heat maps for the individual cell models. The expression patterns of DE genes also indicate gene clusters in the heat maps supported by the row dendrograms.

### 3.2. HUVECs Respond by Upregulating Host Defense Genes

**Figure 4:**
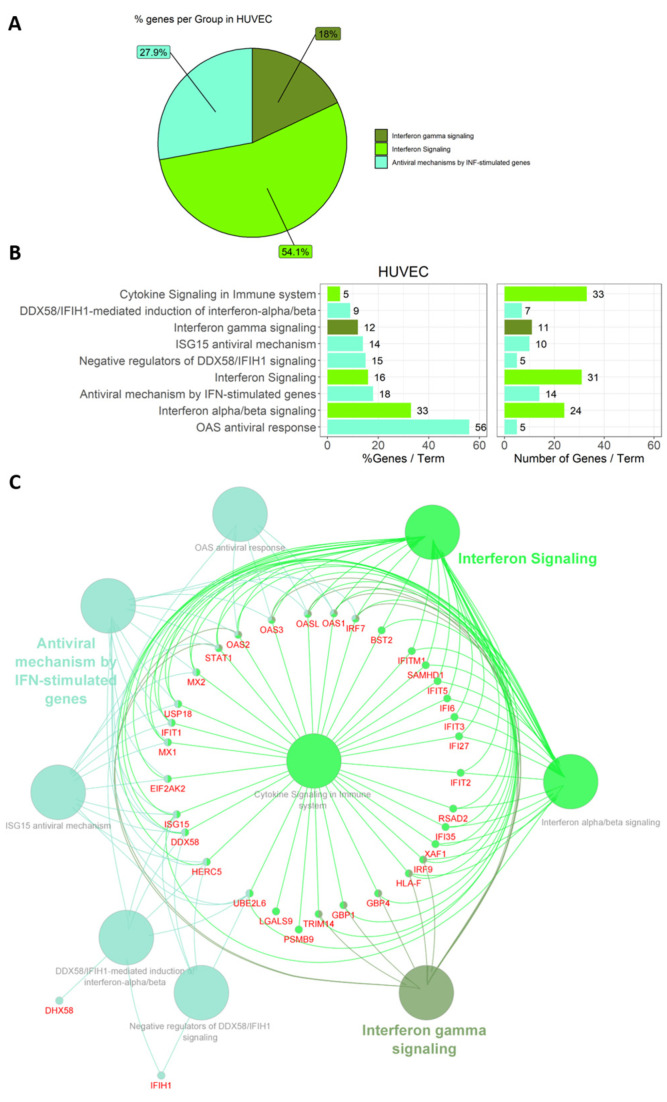
Functional annotation of DE genes in HUVECs. DE genes in HUVECs were assigned to three groups, as depicted in the pie chart (**A**) in three shades of green. Genes were assigned to nine pathway terms, with % genes/term and the number of genes/term mapped separately (**B**). The generated network (**C**) shows the groups (colored), pathway terms (grey), and shared genes (red) according to the functional analysis.

**Figure 5:**
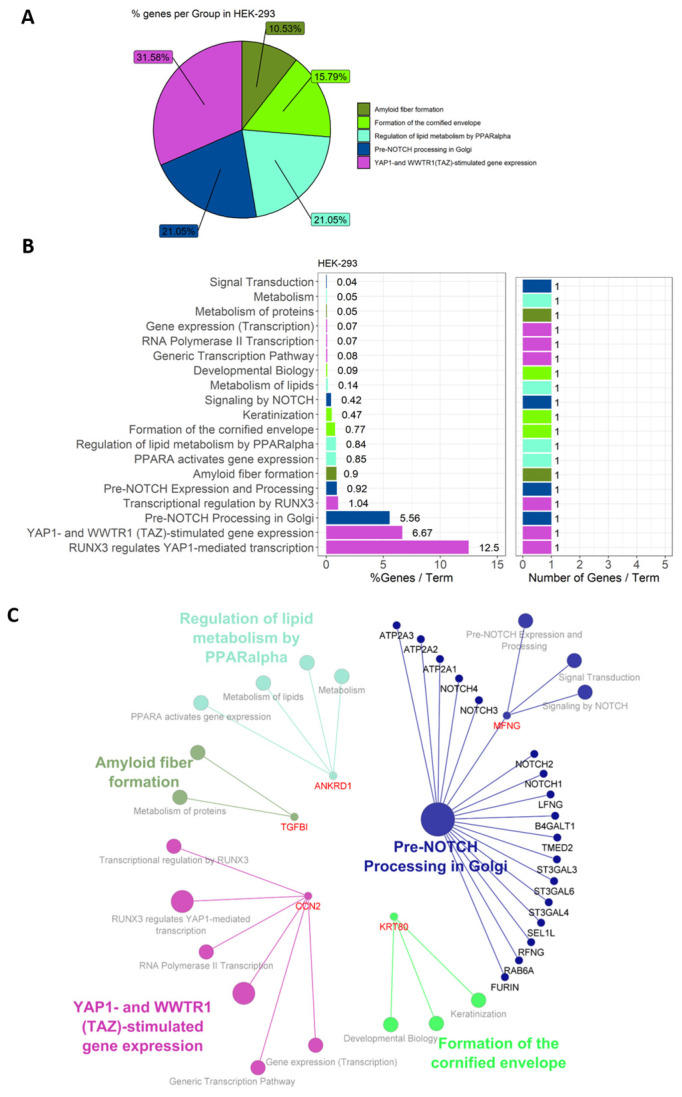
Functional annotation of DE genes in HEK-293 cells. All potential representative pathways are depicted, while only “Pre-NOTCH Processing in Golgi” is significant. DE genes in HEK-293 cells were assigned to five groups, as depicted in the pie chart (**A**). Genes were assigned to 19 pathway terms, with % genes/term and the number of genes/term mapped separately (**B**). The generated network (**C**) shows the groups (color coded), pathway terms (grey), and contributing genes (red) according to the functional analysis.

The identified DE genes were subjected to functional analysis with Cytoscape App ClueGO using reactome pathways for enrichment analysis and visualization in networks. Representative pathways with mapped genes, and the percentage of involvement in the identified groups and terms are depicted for HUVECs in Figure 4 and for HEK-293 cells in Figure 5.

**Figure 6:**
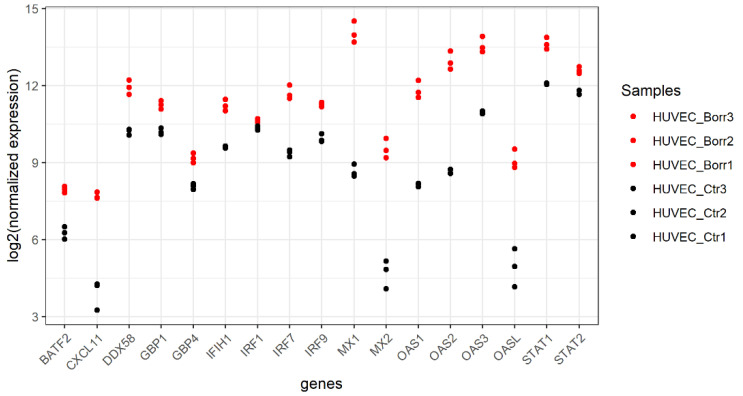
Expression of selected DE genes in HUVECs unexposed or exposed to *B. burgdorferi* strain B31. Basic leucine zipper transcription factor (BATF2), C-X-C Motif Chemokine Ligand 11(CXCL11), retinoic acid-inducible gene-I (RIG-I)-receptor (DDX58), guanylate-binding *proteins* (*GBP*), RIG-I-like receptor (IFIH1), interferon regulatory factors (IRFs), MX proteins that are dynamin-like GTPases, members of the 2′-5′-oligoadenylate synthetase (OAS) protein family, and two Signal Transducer and Activator of Transcription (STAT) factors from the data set are highlighted here. These genes, some of which were also the main players in the functional enrichment analysis used to explain the induced immune response, are clearly shown to be induced in their expression by *B. burgdorferi* exposure. Genes are plotted against log2 (normalized expression) per replicate (1–3) of the different cell treatments. Red dots represent *B. burgdorferi*-exposed cells (Borr), and black dots represent uninfected HUVECs (Ctr).

Four of the 69 significantly DE genes were not annotated. Of the remaining 65, 53 (81.5%) were recognized and included in the functional annotation. These genes were associated with nine representative terms and pathways after p-value significance selection criteria (Figure 4B). They were divided into the following three groups: “interferon–gamma signaling”, “interferon signaling”, and “antiviral mechanisms by IFN-stimulated genes”. “Interferon signaling” was the group with the highest percentage of genes per group (Figure 4A). “Interferon alpha/beta signaling” and “OAS antiviral response” were attributed the highest percentages of genes of a pathway/term, while the number of genes per term varied and most genes were associated with “cytokine signaling in the immune system”, with 33 genes involved in this pathway (Figure 4B). The generated network for HUVECs (Figure 4C) shows the terms as intersection points linked based on a previously determined kappa value. All three groups with assigned pathway terms share the genes STAT1, OAS1, OAS2, OAS3, OASL, and IRF7, as depicted in the circular layout network shown in Figure 4C. In HUVECs, exposure to *B. burgdorferi* results in a pronounced induction of gene expression, as documented in Figure 6 for a selection of genes.

### 3.3. Gene Expression Patterns in HEK-293 Cells Differ Distinctly from HUVECs

**Figure 7:**
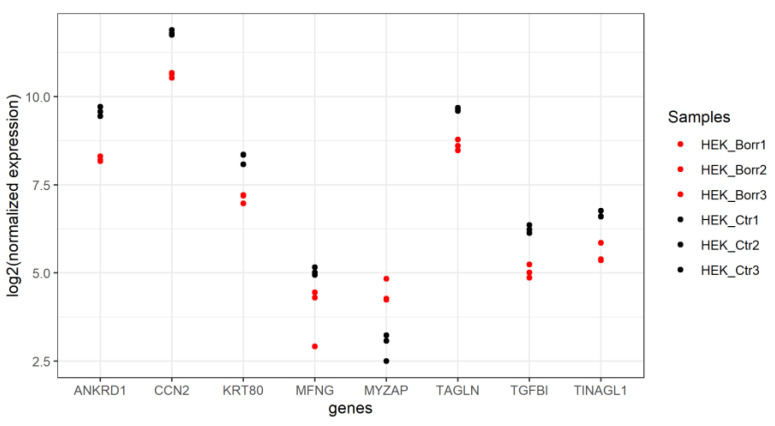
Expression of DE genes in HEK-293 cells unexposed or exposed to *B. burgdorferi* strain B31. Most DE genes in HEK-293 cells are downregulated upon *B. burgdorferi*-exposure. The 8 DE genes code for the following proteins: Ankyrin Repeat Domain 1 (ANKRD1), Cellular Communication Network Factor 2 (CCN2), Keratin 80 (KRT80), O-Fucosylpeptide 3-Beta-N-Acetylglucosaminyltransferase or manic fringe (MFNG), Myocardial Zonula Adherens Protein (MYZAP), Transgelin (TAGLN), Transforming Growth Factor Beta 1 (TGFB1), and Tubulointerstitial Nephritis Antigen-Like 1 (TINAGL1).

Of the eight significantly DE genes in HEK-293 cells, five (62.5%) were functionally annotated and associated with 19 representative terms and pathways after applying general selection criteria (Figure 5). Gens were assigned to the following five groups: “amyloid fiber formation”, “formation of the cornified envelope”, “regulation of lipid metabolism by PPARalpha”, “pre-NOTCH processing in Golgi”, and “YAP1-and WWTR1(TAZ)-stimulated gene expression” (Figure 5A). Because of the short length of the input gene list, every term was associated with only one gene, each of which accounted for a different percentage in the signaling pathway (Figure 5B). Applying Benjamini–Hochberg correction to the pathway analysis, only “pre-NOTCH processing in Golgi” and MFNG were returned as significant. Unlike HUVECs, DE genes in HEK-293 cells were mostly downregulated in response to *B. burgdorferi*, except for MYZAP (Figure 7).

### 3.4. Transcriptional Changes Are Not Reflected by Methylome Changes

**Figure 8:**
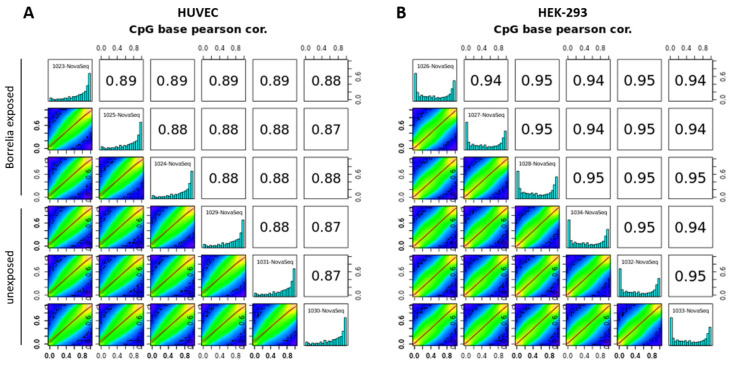
Correlation of methylation across samples. Histograms displaying percentage of methylation per cytosine base for unexposed and *B. burgdorferi*-exposed replicates are shown diagonally for each cell model. Numbers in the top-right panels indicate Pearson correlation values of the pairwise comparison. The panels in the lower left are scatter plots of percentage of methylation for each sample pairing. The settings for sample comparison of unexposed and *B. burgdorferi*-exposed cells in the figures shown here were 20% methylation difference, minimum CpG coverage of 10×, and a *q*-value 0.05 for both HUVECs (**A**) and HEK-293 cells (**B**).

Both cell models exposed to *B. burgdorferi* strain B31 were examined for changes in DNA methylation. A total of 5.7 billion reads were obtained from EM-seq. Reads were aligned (directionally) to the human genome (hg19) and to the pUC19 and lambda genomes using the Dragen methylation pipeline in Illumina BaseSpace. Validation of conversion for methylated spike-in control pUC19 revealed an average CpG methylation of 97.73%, ranging from 95.6 to 98.9%. Unmethylated spike-in control lambda showed an average CpG methylation of 0.66%, with two clear outliers of 1.92% and 3.44% CpG methylation (Supplement File S2). These results are in accordance with the quality controls and confirm that the method worked. An average of 215.47 million reads per sample was used for alignment to the human reference genome, resulting in an average mapping efficiency of 45.23%. The efficiency of mapping to the human genome was relatively low in this study, although FastQC was considered to be good; low mapping efficiency has also been reported for other genomes using Bismark. The conversion metrics for the human genome revealed an average CpG methylation of 69.73%, with methylation almost exclusively associated with CpGs and not CHG or CHH. Histograms of % CpG methylation in the forward and reverse strands were comparable for all samples and showed either high or low percentages of methylation per base (Figure 8). Pearson pairwise correlation analysis between the percentage of methylation profiles across all samples of HUVECs and HEK-293 cells showed strong sample correlation independent of the exposure status (Figure 8). This high correlation of the samples indicates that there were hardly any measurable differences due to *Borrelia* exposure. Presumably due to the primary cell origin, the strong correlation values for HUVECs are slightly lower compared to those for HEK-293 cells in all modified settings for differential methylation analysis. CpG coverage was plotted for both cell models to ascertain the quality of reads from EM-seq (Supplemental Figure S2). A selected CpG coverage of 5 or 10× suggests that the coverage is related to true CpG and not a technical artifact.

Differential methylation analysis testing variable settings of percentage difference and coverage between comparisons and applying two different q-values with MethylKit did not reveal any significant methylation differences between unexposed and *B. burgdorferi*-exposed cells in either cell model (Figure S4). Thus, transcriptional changes are observed but not associated with changes in global DNA methylation, although highly localized differences in DNA methylation below the threshold of detection cannot be excluded.

### 3.5. Changes in Non-Coding RNAs in Response to Borrelia-Exposed HUVECs

Four genes of the DE genes of HUVECs were not annotated with a gene symbol, but they showed a log2FC that was considered significant between *B. burgdorferi*-unexposed and -exposed cells. Manual inspection revealed that these gene loci encode a pseudogene (N-ethylmaleimide-sensitive factor pseudogene 1 (NSFP1), ENSG00000260075.1), a novel transcript (long ncRNA, ENSG00000272512.1), a novel protein (ENSG00000285304.1), and a processed transcript (PDCD6-AHRR readthrough (NMD candidate) (PDCD6-AHRR) ENSG00000288622.1). Both the novel transcript and the processed transcript are RNA genes that belong to the long non-coding RNA (lncRNA) class according to GeneCards (www.genecards.org). Transcription of the novel transcript (lncRNA) was doubled upon *B. burgdorferi* exposure, while PDCD6-AHRR was downregulated in exposed HUVECs.

### 3.6. Overlap of Differentially Expressed Genes between This Study and Other Studies of Borrelia-Exposed Cells

**Figure 9:**
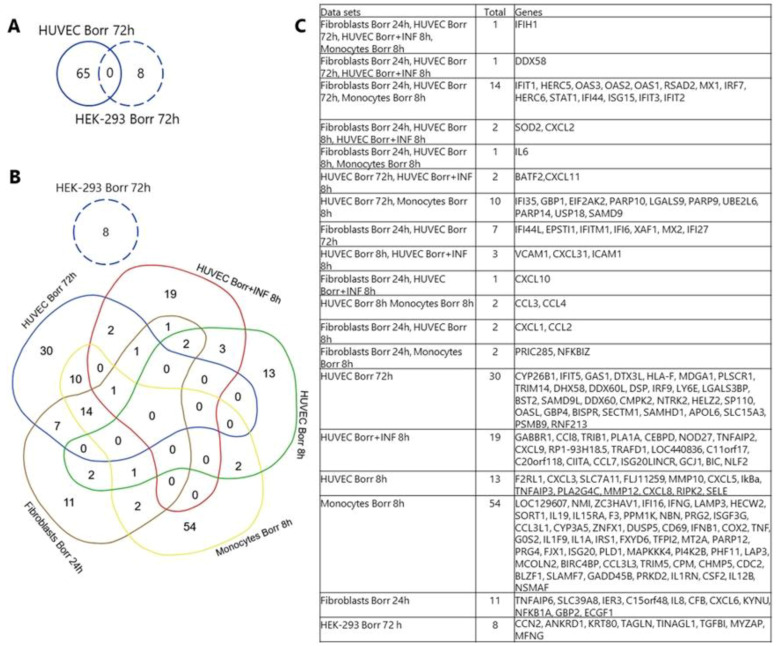
Comparative analysis of gene expression induced by *B. burgdorferi* in different cell types. (**A**) Differentially expressed (DE) genes identified in this study for HUVECs and HEK-293 cells exposed to *B. burgdorferi* for 72 h were compared and showed no overlap in a Venn diagram. (**B**) The data sets are color-coded. Dame et al. (2007) examined the effects of *B. burgdorferi* with an MOI of 10:1 (green) and *B. burgdorferi* (MOI 10:1) in combination with interferon gamma (IFN-γ) (red) on HUVECs after 8 h exposure by microarray. Salazar et al. (2009) exposed monocytes to lysed and live *B. burgdorferi* for 8 h at various MOIs (1:1,10:1,100:1). Genes exclusively or more intensely upregulated by live *B. burgdorferi* (yellow) were included in the present comparative study. Meddeb et al. (2016) used fibroblasts which exposed to *B. burgdorferi* and two other pathogenic *Borrelia* strains at an MOI 100:1 for 24 h (brown) and subjected to microarray analysis. Consistent DE genes in response to the three pathogenic were considered for this comparative study. The data sets in blue represent the gene set of the present study, where HUVECs (solid blue line) or HEK-293 cells (dashed blue line) were exposed to *B. burgdorferi* for 72 h at an MOI of 50:1. Our HUVEC data set (solid blue) shows no overlap with HUVECs exposed to *B. burgdorferi* for 8 h (green). HUVECs showed some gene overlap with the others studies, while no shared DE genes were found between HEK-293 cells and the other data sets. The table (**C**) provides the gene names.

An integrative computational approach was used to compare the results of this study with relevant previously published transcriptome data. Figure 9 shows Venn diagrams and a table of overlaps and unique gene expression profiles for the two cell models exposed to *B. burgdorferi* B31 in this study versus other studies of host cell–pathogen interaction.

Dame et al. (2007) studied the effects of *B. burgdorferi* at an MOI of 10:1 and *B. burgdorferi* (MOI 10:1) in combination with interferon gamma (IFN-γ) on HUVECs after 8 h exposure by microarray [59]. Dame et al. (2007) found that *B. burgdorferi*, together with IFN-γ, induced more genes in HUVECs. Salazar et al. (2009) exposed monocytes to lysed and live *B. burgdorferi* for 8 h at various MOIs (1:1, 10:1, 100:1) [53]. Genes that are upregulated exclusively or more strongly by live *B. burgdorferi* than by dead bacteria were included in the comparative study presented here, as we used live bacteria. In a study by Meddeb et al. (2016), fibroblasts were exposed to *B. burgdorferi* and two other pathogenic *Borrelia* strains at an MOI of 100:1 for 24 h and subjected to microarray analysis [40]. Genes consistently differentially expressed in response to the three pathogenic *Borrelia* species were considered for this comparative study.

The data show no overlap between DE genes found in HUVECs and HEK-293 cells exposed to *B. burgdorferi* for 72 h (Figure 9A). HUVECs showed some overlap with DE genes derived from transcriptome data from HUVECs, monocytes, and fibroblasts exposed to *B. burgdorferi* for other time periods (Figure 9B). Four genes of our HUVEC data set (solid blue line) overlap with HUVECs exposed to *B. burgdorferi* in combination with IFN (red data set in Figure 9B). A total of 25 genes are shared with monocytes exposed to *B. burgdorferi* for 8 h (yellow), and 26 genes are shared with fibroblasts exposed to *B. burgdorferi* for 24 h (brown) and our HUVEC data. In contrast, HEK-293 cells had no genes concordant with these data sets.

## 4. Discussion

**Figure 10:**
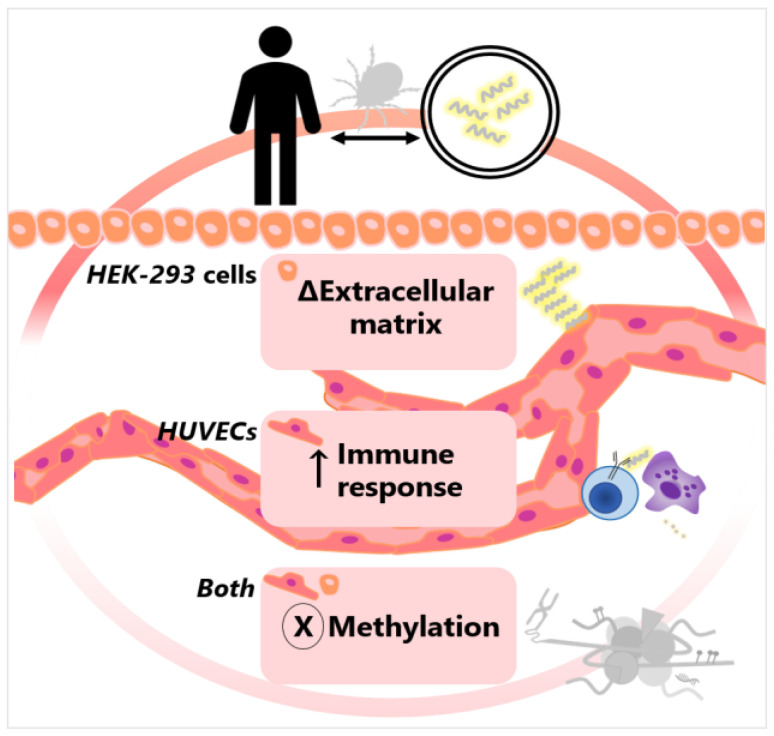
Summary of findings in human cells exposed to *Borrelia burgdorferi*.

Many questions exist about the interactions between the *Borrelia* pathogen and the host cell that results in Lyme disease. Here, we have focused on representative human cell models with the goal of achieving a more detailed understanding of the interactions between *B. burgdorferi* and human cells in a time frame consistent with committed cellular responses but long enough to encompass the internalization of *Borrelia* in immune and other cells and robust cellular responses, genetic and epigenetic characteristic of early infection. We investigated changes in gene transcription, including non-coding RNA genes, and DNA methylation after 72 h of pathogen exposure. Previous transcriptional studies have used microarray or RNA-seq for blood cells [53,54,79], fibroblasts [37,40,80], endothelial [48,59] and neuronal cells [81], spleen cells [52], and breast cancer cell lines [42], finding transcriptional changes after exposure to *B. burgdorferi*. Our study reinforces the occurrence of transcriptional changes induced by *B. burgdorferi*. These occurred to a much greater extent in HUVECs than in HEK-293 cells, and these cell models showed different spectra of genes and effects on gene expression. These findings are summarized in Figure 10.

### 4.1. Immune Response of HUVECs

The cellular origin differs between the two cell models used in our study. HUVECs are a human primary endothelial cell line. Primary cells are generally considered to be more physiologically relevant than immortalized cell lines. Furthermore, endothelial cells are the first line of defense against infectious agents. Endothelial cells line the body’s blood circulatory system and are immunologically significant due to their production of pro-inflammatory signaling molecules, as well as the selective permeability of immune cells into tissues [82,83]. They are also crucial for nutrient supply and removal of waste products [84]. Thus, endothelial cells, as a cellular barrier between blood and tissues, inevitably come into contact with *Borrelia* sp. during host infection. This occurs either during local infection in the skin or during the spread of infection in the body. Endothelial cells can act as a sensor for the pathogen or provide a replicative niche [83].

In our transcriptome study, the cellular immune response in HUVECs was particularly pronounced after B. burgdorferi exposure, highlighting its cellular role as an important nexus mediating between the pathogen and host. Pathways enriched for pro-inflammatory cytokines like interferon gamma (IFN-γ) signaling indicate linkage of innate and adaptive immune response that can recruit immune cells to the *B.* burgdorferi infection side in vivo [85,86]. IFN-γ was one of the major cytokines found in erythema migrans, one of the early signs of Lyme disease [87,88]. Dame et al. (2007) found that the simultaneous stimulation of endothelial cells with B. burgdorferi and IFN-γ led to more dramatic transcriptional changes and induction of chemokines [59]. High INF levels have also been found in sera from Lyme disease patients; in patients with late or chronic Lyme disease, these high INF levels indicate a persistent immune response and possible continued infection [59,87]. In our study, cytokine signaling in the immune system and INF signaling (INF-α, -β, -γ) were induced after 72 h, thus the overall inflammatory picture of early Lyme disease was reproduced in our study in vitro in HUVECs.

Although Dame et al. (2007) similarly detected inflammatory response in HUVECs, the majority of affected genes were different compared with our results. Surprisingly, there is no overlap of the *B. burgdorferi*-induced genes in HUVECs incubated for 8 h [59] and the DE genes we found in HUVECs incubated with *B. burgdorferi* for 72 h in this study (Figure 9). In the Dame et al. (2007) microarray study, *B. burgdorferi*, IFN-γ, and both showed strong induction of chemoattractants (CCL7, CCL8, CX3CL1, CXCL10, CXCL2, and CXCL9) and adhesion molecules (ICAM-1 and VCAM-1) in HUVECs after 8 h [59]. Similarly, fibroblasts exposed to three species of *B. burgdorferi* sensu lato for 24 h showed strong induction of chemoattractant molecules [40]. However, for HUVECs induced for 8 h with *B. burgdorferi* in combination with IFN-γ [59], there are four common genes (DDX58, IFIH1, BATF2, and CXCL11) compared to the present experiments in HUVEC (Figure 9). DDX58 is a retinoic acid-inducible gene-I (RIG-I) receptor. The IFIH1 gene encodes MDA5, a RIG-I-like receptor. Both are crucial for recognition of pathogens in cytosol and induce IFN-stimulated genes (ISGs), type I IFN, and pro-inflammatory cytokines [89]. Both genes are also induced by *B. burgdorferi* in fibroblasts [40] and monocytes [53] exposed for 4 to 24 h. Basic leucine zipper transcription factor (BATF2) controls immune cell differentiation and macrophage activation [90]. CXCL11 is known to be induced by IFN-γ, is crucial for activated T-cell recruitment, and has been identified as important for the balance of angiogenesis and angiostasis [91]. These gene products are important connectors between the innate and adaptive immune systems, relying largely on IFN signaling; therefore, they may play an important role in the early phase of *Borrelia* infection.

A particularly interesting result is that endothelial HUVECs expressed increased levels of STAT1, OAS1, OAS2, OAS3, OASL, and IRF7 after 72 h *B. burgdorferi* exposure; these genes were present in all identified pathway groups in the functional analysis. An essential component of the IFN signaling pathway via JAK-STAT is the Signal Transducer and Activator of Transcription 1-alpha/beta (STAT1). STAT1 is either phosphorylated or associated with other factors (STAT2, IRF9), which enables the cytosol-to-nucleus transposition and expression of ISGs that are crucial for response to pathogen stimuli [92]. Among the triggered ISGs are the 2′-5′-oligoadenylate synthetase (OAS) protein family, which comprises OAS1, OAS2, OAS3, and OAS-like (OASL) [93]; all four were found to be induced by exposure to *B. burgdorferi* in our study. OAS proteins recognize double-stranded RNAs and are thought to have mainly antiviral functions by activating RNase L to eliminate pathogen load through RNA degradation [94]. However, recently, OAS proteins were also found to be induced by tuberculosis bacteria in macrophages and to positively affect the expression of TNF-α and IL-1β to support host defense mechanisms [95]. *Borrelia* internalization and cellular processing have been well studied in macrophages [96]. Different pattern recognition receptors in the endosomal compartment recognize cell-invasive pathogens such as bacteria or viruses, and the downstream adaptor proteins induce interferon regulatory factors (IRFs), including IRF7, that control and promote the immune response by inducing more ISGs in the nucleus [97,98]. Another group of ISGs is guanylate-binding proteins, among which GBP1 and GBP4 were found to be significantly expressed in response to *B. burgdorferi* in our study (Figure 6). In addition to their activity against viruses, these proteins have been similarly reported to be active against intracellular bacteria by boosting innate immunity and helping to control tissue damage [99]. GBP1 has a recognition function for cytosolic Gram-negative bacteria such as salmonella or shigella and induces the recruitment of additional GBPs and caspase-4, targeting the degradation of the pathogen [100,101]. Complementing the groups of ISGs found in this study are proteins MX1 and MX2. They are dynamin-like GTPases that are induced through the INF signaling pathway via JAK-STAT [102]. GBP1, MX1, OAS1, OAS2, OAS3, and IRF7 are among 25 genes shared by monocytes exposed for 8 h to *B. burgdorferi* [53] and DE genes found in this study in HUVECs. ISGs, like IFIT, OAS1-3, MX1, MX2, and IRF7, were among the 23 genes shared by fibroblasts exposed to *B. burgdorferi* for 24 h and HUVECs in this study. The different suites of genes found to be differentially regulated by exposure to *B. burgdorferi* in our study compared to others emphasize the temporal induction of key factors in the immune response, which is crucial for defense against pathogens in different cell types. Similarly, upregulation of immune-effector molecules such C-X-C and C-C motif chemokine family members and genes and pathways associated with infection, inflammation, and cancer was found in breast cancer and normal-like cell models [42].

The cellular response we found in this study in HUVECs is primarily aimed at actively eliminating pathogens within the cell. *Borrelia* occur both extracellularly and intracellularly [41,66,103,104]. Karvonen et al., (2021) found some internalized *B. burgdorferi* in human cells after 24 and 72 h of exposure [66]. In the same study, invasion of two non-immune cell lines by *B. burgdorferi* was observed in different cellular compartments, although not the lysosomes, suggesting that *B. burgdorferi* has strategies for intracellular survival [66].

### 4.2. The Cellular Response of HEK-293 Cells Suggests ECM Remodelling That Could Promote Bacterial Survival

The properties of HEK-293 cells, being derived from a human embryo, are complex [105]. The exact origin of the cells from the embryo is unclear, and the characteristics of epithelial cells, mesenchymal cells, and neuronal cells are attributed to these cells [106]. All three of these cell types are also resident in the dermis and are involved in interactions between *Borrelia* and the host, so immortalized HEK-293 cells are relevant as a cell model in this study. HEK-293 cells possess a repertoire of receptors for pathogen recognition, such as plasma membrane-residing Toll-like receptor (TLR) 5, endosome-associated TLR3, and cytosolic nucleotide-binding oligomerization domain NOD1; however, importantly, they lack surface-expressed TLR2 [107,108,109]. TLR receptors induce an inflammatory signaling cascade through the NF-κb or IRF pathway. In monocytes, TLR2 and TLR8 contribute to effective degradation of the *Borrelia* pathogen in the phagosome [110]. In vivo, TLR2 deficiency in mice has been reported to result in persistently increased *Borrelia* loads in tissues [111], and genetic variation in TLRs corresponds with certain infectious disease risks [112]. Therefore, studying host response to *Borrelia* in cells with some but not other receptors of pathogen recognition could contribute to understanding strategies of bacterial survival within the host cell.

Integrins mediate cellular signaling and communication from the extracellular environment and, as such, act as recognition molecules for *Borrelia* [41,113,114]. Interaction between *Borrelia* and the host includes binding and degradation of the extracellular matrix (ECM) through the induction of metalloproteases [115,116], the induction of Src family kinases, or the reorganization of actin filaments [41]. All these mechanisms allow for host-cell invasion and the spread of the pathogen through host tissues. In our study, we found that TGFB1, TINAGL1, and CCN2 were downregulated in HEK-293 cells after *B. burgdorferi* exposure. All of these gene products are thought to be responsible for or contribute to the binding and organization of the ECM. Transforming Growth Factor Beta 1 (TGFB1) encodes a secreted ligand of the TGF-β (transforming growth factor-beta) protein family. As a latent TGF-β binding protein complex, these proteins interact with fibrillin of the ECM of cells [117]. TGF-β signaling contributes to a multitude of cellular processes, including extracellular matrix remodeling [118]. Tubulointerstitial nephritis antigen 1 (TINAGL1) is an abundant matricellular renal protein in the basal layer of the ECM [119] and plays a modulatory role through interaction with ECM elements such as fibronectin and integrins [120]. One member of the Cyr61/CTGF/NOV (CCN) protein family, CCN2, also termed connective tissue growth factor (CTGF), plays a role in the survival of neuronal and other cells [121] and the normal production of vascular basement membranes [122]. This matricellular protein interacts with integrins, low-density lipoprotein receptor-related proteins, growth factors, and ECM components [122], contributing to ECM function. Downregulation of these gene products in HEK-293 cells upon *B. burgdorferi* exposure, all with functions involving ECM interaction, could favor bacterial survival and invasion. The absence of TLR2 in HEK-293 cells, one of the recognition receptors of *Borrelia*, also suggests that *Borrelia* are unrecognized and survive.

Basic cytokeratin KRT80 is a cytosolic filament that generally has a structural function in epithelia and interacts with cell-adhesive complexes, and its expression is modulated during cell differentiation [123]. KRT80 was downregulated by half in *B. burgdorferi*-exposed cells relative to control cells, which could suggest complex *B. burgdorferi*-induced changes interfering with the cellular structure and signaling. The only gene in HEK-293 cells that was induced upon *B. burgdorferi* exposure encodes the Myocardial Zonula Adherens Protein (MYZAP or MYOZAP). In addition to detection in cardiac myocytes [124] and endothelial cells [125], the presence of MYZAP has been demonstrated in adherens junctions in various epithelia, including cancer cells [126]. The presence of MYZAP in cell–cell contact structures in epithelia is thought to be essential for proper scaffold function, which also requires actin filaments and actin-binding proteins [126]. It remains to be elucidated what role upregulation of MYZAP plays in the presence of *B. burgdorferi*.

O-Fucosylpeptide 3-Beta-N-Acetylglucosaminyltransferase or manic fringe (MFNG) is downregulated in *B. burgdorferi*-exposed HEK-293 cells in comparison to unexposed cells. MFNG participates in the “pre-NOTCH signaling in Golgi” pathway. NOTCH signaling is a highly conserved signaling pathway that contributes to differentiation, proliferation, and apoptosis depending on the cell context [127]. NOTCH receptor precursors (pre-NOTCH) are post-translationally modified by the addition of O-glucose, O-fucose, and O-N-acetylglucosamine (GlcNAc) by members of the fringe family (lunatic, manic, and radical fringe) in the Golgi membrane [128]. Interfering with the glycosylation machinery alters signal recognition and downstream cellular processes, which might benefit *Borrelia* survival, potentially by suppressing apoptosis.

### 4.3. B. burgdorferi as an Instigator of Epigenetic Changes

We wished to determine whether the observed transcriptional changes induced by *B. burgdorferi* were due to underlying global DNA methylation changes. We did not find global DNA methylation change; however, this does not exclude localized DNA methylation changes below the limit of detection or over longer (or shorter) time periods. It is possible that a 72 h exposure was not long enough to induce de novo DNA methylation in the human cells; prolonged co-culture of cells with *B. burgdorferi*, representing the persistence of the host infection, might induce epigenetic changes. The contributions of other epigenetic mechanisms like histone modifications or non-coding RNAs (ncRNAs) remain to be explored.

However, in our HUVEC dataset, we found potential evidence of epigenetic modification by identifying pseudogene NSFP1, a novel transcript (long ncRNA (lncRNA)), and PDCD6-AHRR (lncRNA) as significantly differentially expressed. The as yet unidentified function of pseudogene NSFP1 could be in the form of a protein or ncRNA, or it could involve regulation of the 3D structure of DNA and its accessibility [129]. These gene products, in the form of long or short ncRNAs, could impact the transcriptional machinery and gene function, with pleiotropic effects on cellular signaling [130]. The deregulation of lncRNAs and their interaction with microRNAs have been described in the literature to occur in the context of infectious diseases [131]. The influence of ncRNAs on host defense signaling has been described in the context of bacterial and viral pathogens, some of which are beneficial for pathogen survival in the host [131]. The function of these ncRNAs in the cell and the consequences of altered expression during *B. burgdorferi* infection remain to be further explored. Genome-wide mapping of the *B*. *burgdorferi* transcriptome identified numerous putative ncRNAs [132]. These extracellular dsRNAs or intracellular dsRNAs could potentially trigger “viral mimicry” signaling through INF, also potentially inducing epigenetic changes in the host cell.

### 4.4. Ticks as an Instigator of Epigenetic Changes

In nature, in addition to the tick-borne pathogens introduced by ticks, tick feeding itself injects tick saliva into the host, and tick saliva is highly complex [133]. In addition to biologically active molecules in tick saliva with anticoagulant, antiplatelet, vasodilator, anti-inflammatory, and immunomodulatory effects and the potential to influence the epigenome, tick saliva transmits miRNAs capable of affecting host gene expression via epigenetic modulation [134]. Another interesting component of tick saliva is tick histone protein H4, a protein identical to human histone H4 [38]. Best known for its structural role in chromatin, tick histone H4 also has an antimicrobial effect on skin commensal bacteria but not on *B. burgdorferi* [38]. Substitution of human with tick histone 4 is one possible avenue for modulation of the host epigenetic machinery. Similarly, the heat-shock proteins found in tick saliva [135], which are immunogenic proteins, can also be epigenetic modulators.

## 5. Conclusions

In this study, we documented a cellular immune response in our endothelial cell model of HUVECs in response to exposure to *B. burgdorferi*. This response was present at 72 h and is common to other cell types and at other time points. Our epithelial cell model of HEK-293 cells demonstrated changes in the expression of genes encoding ECM-resident or -interacting proteins. Changes in DNA methylation were not detectable in these cells; however, ncRNAs were upregulated in response to infection, suggesting that other epigenetic mechanisms may underlie these changes in gene expression.

## Data Availability

All NGS data underlying this study were deposited on database servers. RNA-seq data are accessible through NCBI’s Gene Expression Omnibus (GEO) under super-series accession number GSE194294. EM-seq data are accessible as Sequence Read Archive (*SRA*) data under accession number PRJNA800079.

## Supplementary Materials

The following supporting information can be downloaded at: https://www.mdpi.com/article/10.3390/genes15081010/s1. Figure S1: Relative log expression (RLE) plots. Boxplots show RLE for transcriptome data obtained from human cell models of HUVECs and HEK-293 cells unexposed (Ctr) or exposed (Borr) to *B. burgdorferi* strain B31 for 72 h. Analyses were performed for individual cell types (A, B) and across cells (C). Experiments were performed in triplicate, as indicated by the sample names. RLE plots were generated with R package EDASeq and were used to verify data normalization with DeSeq2. Box plots demonstrate a small deviation from zero, representative of the ideal distribution of normalized counts for all comparisons in this study. Figure S2: Heat map of differentially expressed (DE) genes in HUVECs. The cell model was unexposed (Ctr) or exposed to *B. burgdorferi* strain B31 (Borr) for 72 h in triplicate (as indicated by sample names). DeSeq2 was used to normalize the RNA-seq data and to analyze gene expression by comparing exposure conditions (Ctr vs. Borr). The list of genes was filtered for p_adj_ < 0.05 and log2FC ≥ |1|, reducing the list of samples to 69 gene loci. The scaled log2, mean-centered expression per replicate, and condition in the significant DE genes of HUVECs are shown. The column-wise dendrogram supports clustering according to exposure (red = Borr, black = Ctrl). Figure S3: Heat map of differentially expressed (DE) genes in HEK-293 cells. The cell model was unexposed (Ctr) or exposed to *B. burgdorferi* strain B31 (Borr) for 72 h in triplicate (as indicated by sample names). DeSeq2 was used to normalize the data and to analyze gene expression by comparing exposure conditions (Ctr vs. Borr). The list of genes was filtered for p_adj_ < 0.05 and log2FC ≥ |1|, reducing the list of samples to 8 significant DE genes. The scaled log2, mean-centered expression per replicate, and condition in the significant DE genes of HEK-293 cells are shown. The column-wise dendrogram supports clustering according to exposure (red = Borr, black = Ctrl). Figure S4: Correlation of methylation across samples with different analytical settings. Correlation plots for HUVECs (top line) and HEK-293 cells (bottom line) were generated with MethylKit in Illumina BaseSpace. Histograms displaying percentage of methylation per cytosine base for unexposed and *B. burgdorferi*-exposed replicates are shown diagonally per square. Numbers in the top-right panels indicate Pearson correlation values of the pairwise comparison of the samples, all of which are above 0.7, indicating a strong correlation. The panels in the lower left are scatter plots of percentage of methylation for each sample pairing. The settings for sample comparison of *B. burgdorferi*-unexposed and -exposed cells were varied between 10 and 25% methylation difference, with minimum CpG coverage of 5 or 10×, and a *q*-value cut-off of 0.01 or 0.05 for both HUVECs and HEK-293 cells. Figure S5: Observed coverage of CpGs after EM-seq in unexposed and *B. burgdorferi*-exposed human cells. DNA was sheared to 300 bp using a sonicator (E210, Covaris) and used as input for EM-seq on a Novaseq 6000. For quality control of the methylome analysis, the CpGs covered in HUVECs (A) and HEK-293 cells (B) unexposed and exposed to *B. burgdorferi* were plotted against observed sequencing depth. A chosen CpG coverage of 5× or 10× suggests confidence in real CpG and not a technical artifact. Supplement File S1: RNA-seq_statistics. Supplement File S2: EM-seq_statistics.
