# Supplementary material for: Changes in the Transcriptome and Long Non-Coding RNAs but Not the Methylome Occur in Human Cells Exposed to Borrelia burgdorferi
Source: Genes (Basel). 2024 Aug 1;15(8):1010. doi: 10.3390/genes15081010 (PMC11353914; doi:10.3390/genes15081010)
Supplement: Supplementary file 1 [file genes-15-01010-s001.zip › genes-3093870-supplementary.pdf]

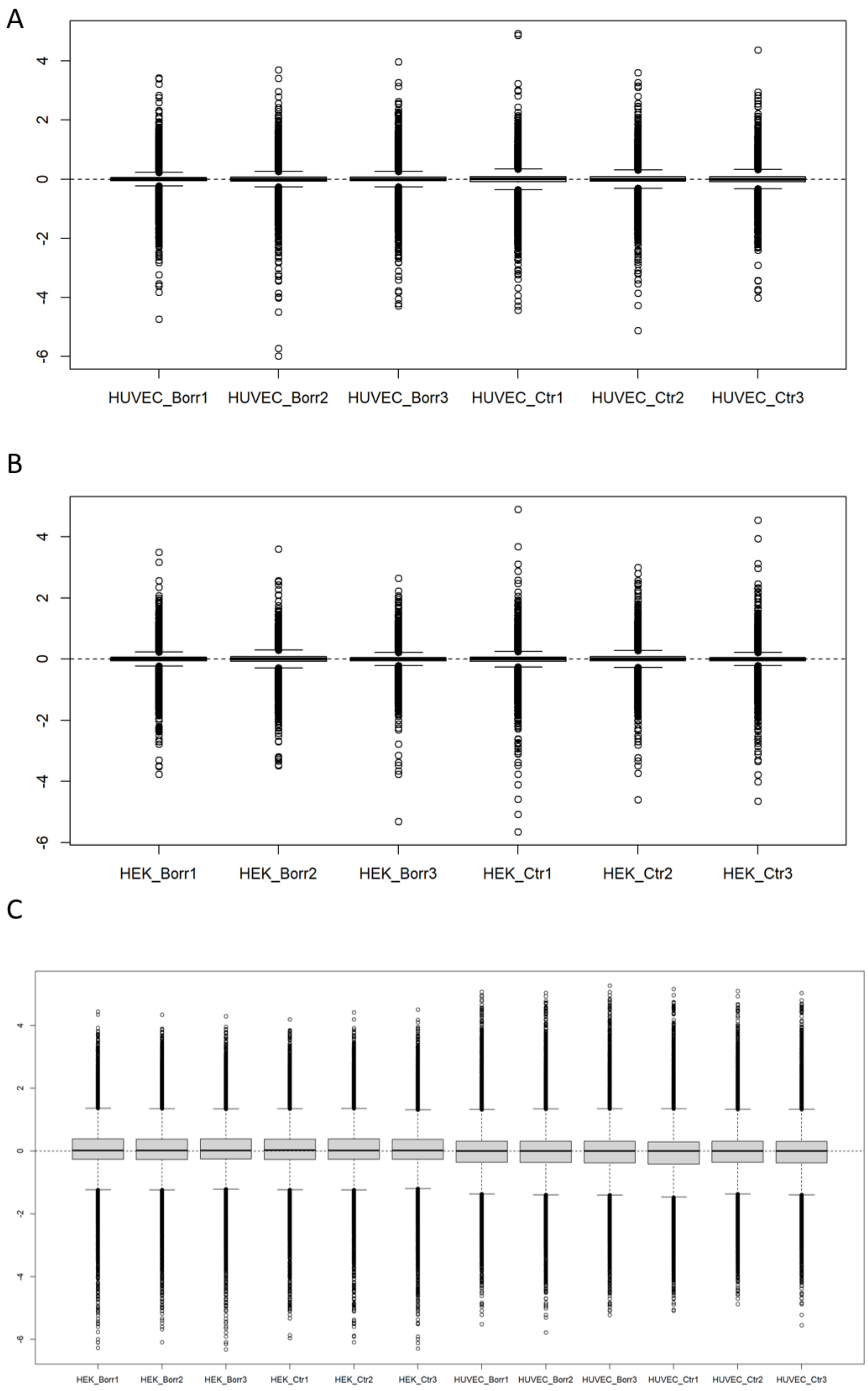

Figure S1: Relative log expression (RLE) plots.

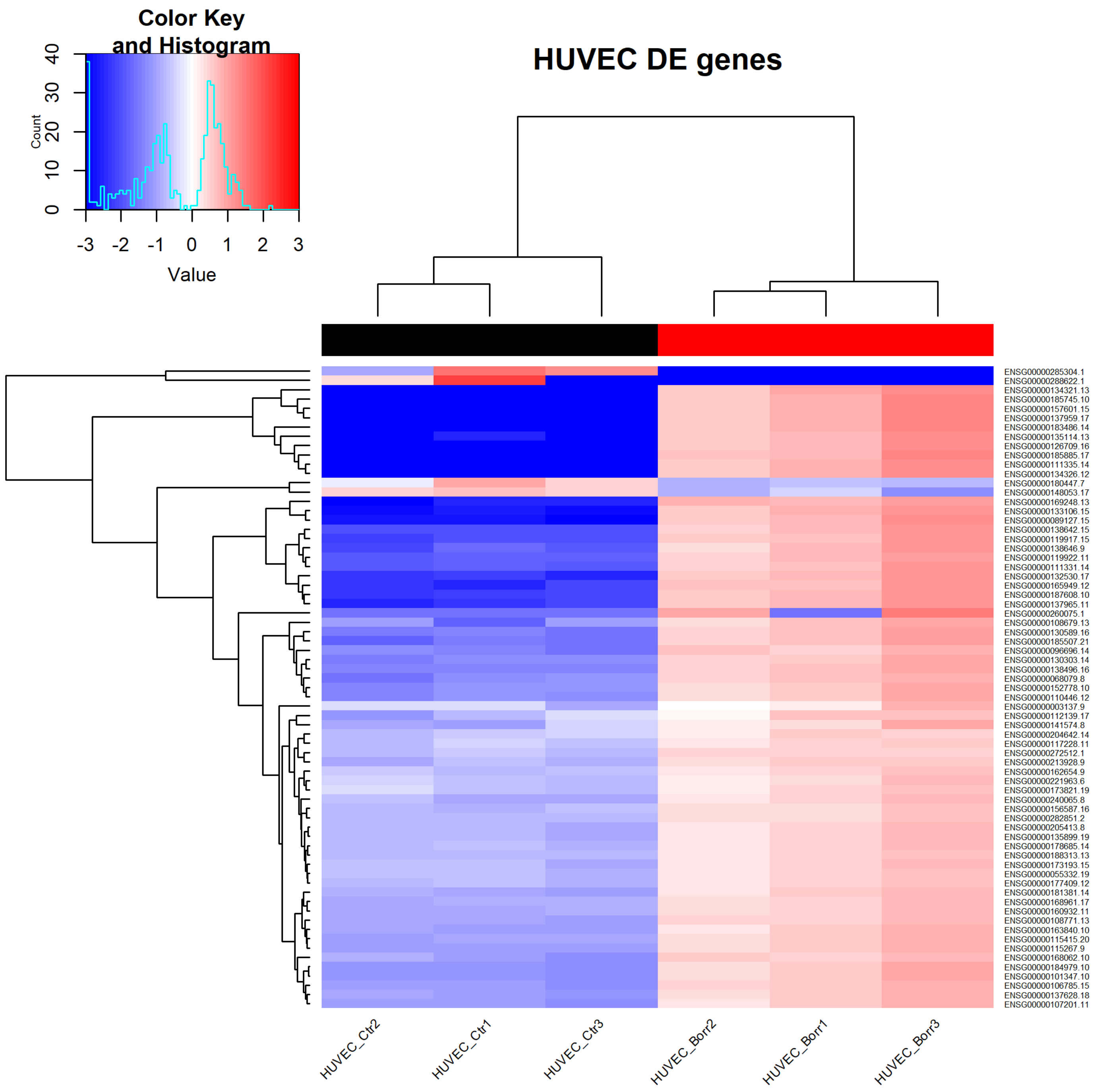

Figure S2: Heat map of differentially expressed (DE) genes in HUVECs.

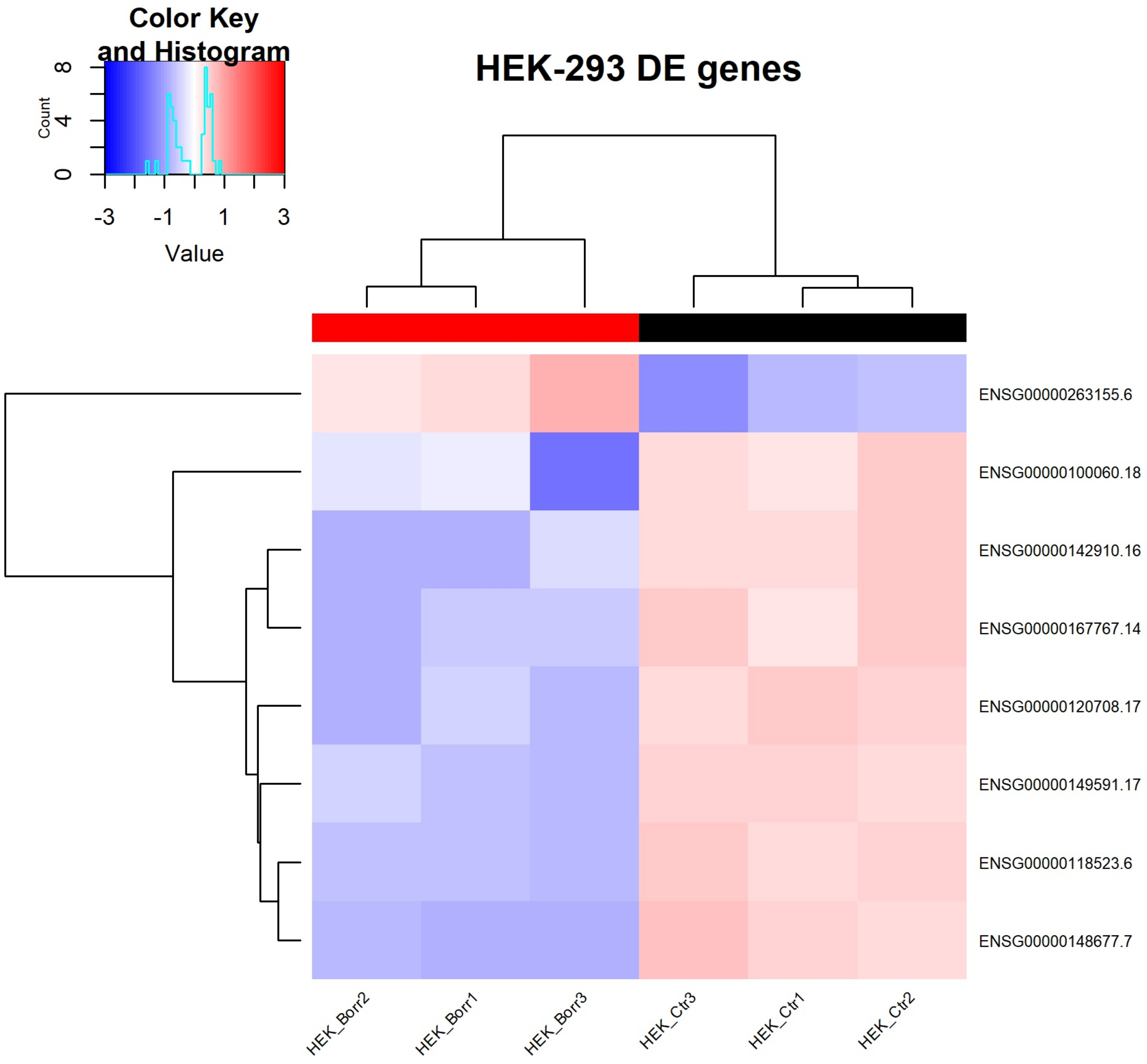

Figure S3: Heat map of differentially expressed (DE) genes in HEK-293 cells.

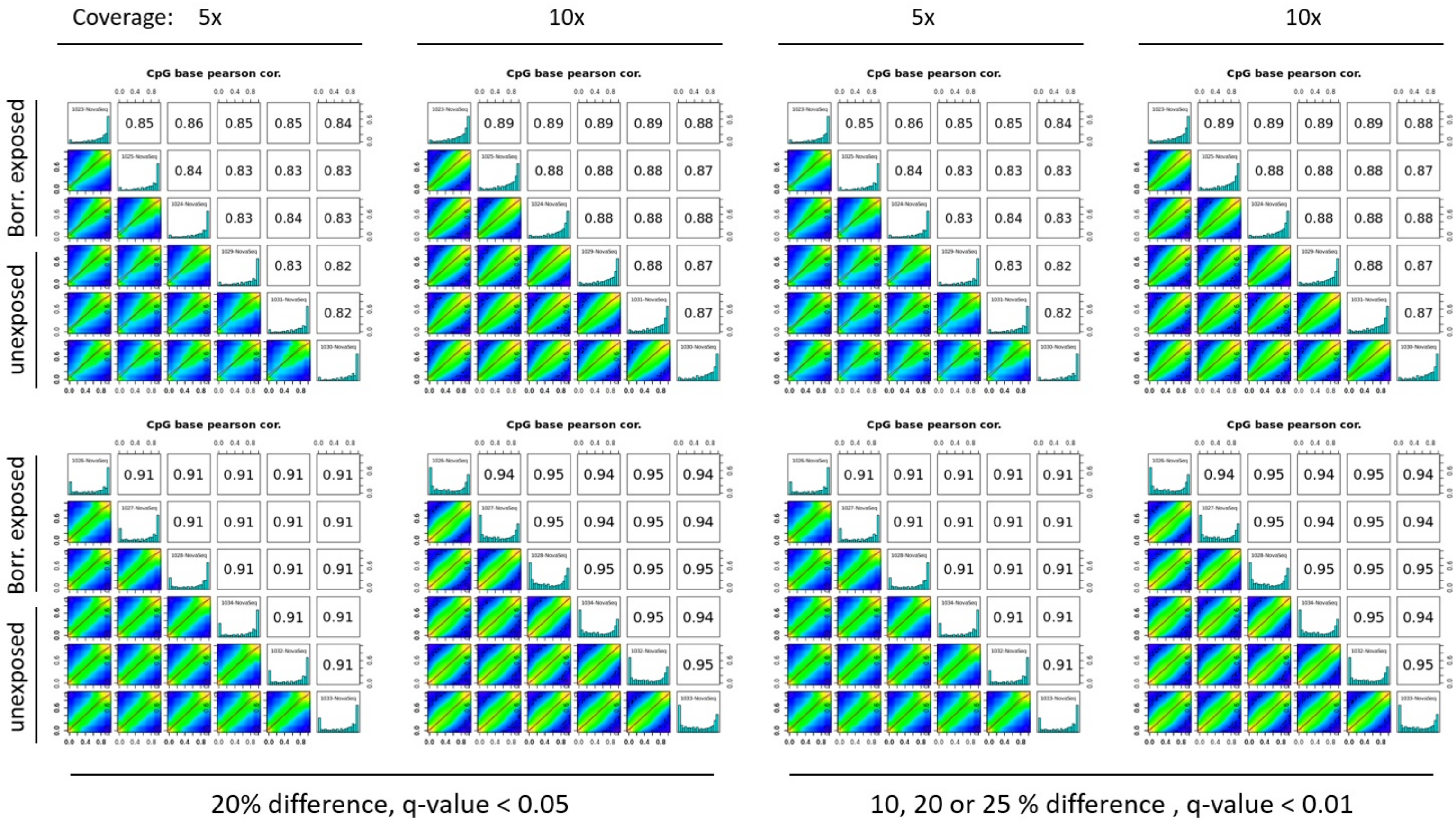

Figure S4: Correlation of methylation across samples with different analytical settings.

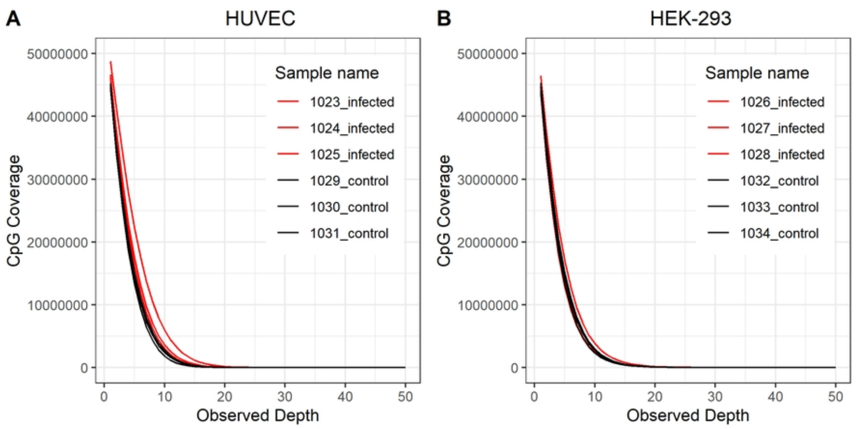

Figure S5: Observed coverage of CpGs after EM-seq in unexposed and B. burgdorferi-exposed human cells.

Supplemental File S1

RNA-seq Statistics:

Sample	Raw reads (total)	total reads after trimming	mapped reads (%)	pct_coding	pct_UTR	pct_intronic	pct_intergenic
AB25	93851724	88911124	98,05	0,577259	0,29183	0,07224	0,058671
AB26	82943343	78914826	98,16	0,576379	0,289496	0,073006	0,061118
AB27	102005948	97035408	98,28	0,582176	0,289642	0,070441	0,057741
AB28	86234932	81554946	97,61	0,556647	0,283456	0,085513	0,074385
AB29	93715989	88548032	97,81	0,568006	0,283725	0,074776	0,073492
AB30	96339069	90954996	97,65	0,562402	0,283298	0,083683	0,070617
AB31	76317337	72576384	98,07	0,587556	0,290902	0,064259	0,057283
AB32	88077465	41820456	98,12	0,572148	0,287773	0,078167	0,061911
AB33	99437002	94586884	98,23	0,568754	0,300233	0,068306	0,062706
AB34	78379041	73699298	97,61	0,556164	0,283103	0,083987	0,076746
AB35	86297812	81272512	97,73	0,571073	0,277433	0,080108	0,071386
AB36	91222004	42888706	97,74	0,565911	0,281879	0,080623	0,071588

Supplement File S2:

Enzymatic Methylation Sequencing- Statistics

Sample template:

1023	AB13-Huvec infected with Borrelia 72h_R1
1024	AB14-Huvec infected with Borrelia 72h_R2
1025	AB15-Huvec infected with Borrelia 72h_R3
1026	AB16-Hek infected with Borrelia 72 h_R1
1027	AB17-Hek infected with Borrelia 72 h_R2
1028	AB18-Hek infected with Borrelia 72 h_R3
1029	AB19-Huvec control 72 h_R1
1030	AB20-Huvec control 72 h_R2
1031	AB21-Huvec control 72_R3
1032	AB22-Hek control 72 h_R1
1033	AB23-Hek control 72 h_R2
1034	AB24-Hek control 72 h_R3

Human Genome

Sample ID	Total PE reads	% PE reads used	Total reads (R1&R2)	Alignment rate	% C's in CPG methylated	% C's in CHG methylated	% C's in CHH methylated
1023	293899737	134590465	269180930	45,9	74,12	0,25	0,23
1024	254750937	116101876	232203752	45,57	73,97	0,2	0,19
1025	246833876	110586772	221173544	44,8	73,39	0,2	0,19
1026	218842948	98997667	197995334	45,24	65,82	0,016	1,4
1027	216451056	98750779	197501558	45,62	65,66	0,92	0,82
1028	258687374	117495728	234991456	45,42	66,32	3,21	3,85
1029	231908301	103861315	207722630	44,79	73,85	0,23	0,21
1030	223595697	98407969	196815938	44,04	73,8	0,21	0,19
1031	237301449	108088633	216177266	45,55	73,67	0,2	0,19
1032	229815714	104123685	208247370	45,31	65,71	0,2	0,18
1033	229483404	103756706	207513412	45,21	65,44	0,39	0,35
1034	216516303	98062029	196124058	45,29	65,06	0,24	0,22

PUC 19

Sample ID	Total PE reads	%PE reads used	% C's in CPG methylated
1023	293899737	213	95,61
1024	254750937	151	97,74
1025	246833876	181	98,45
1026	218842948	142	98,7
1027	216451056	164	97,54
1028	258687374	150	98,85
1029	231908301	129	97,88
1030	223595697	158	97,96
1031	237301449	189	96,83
1032	229815714	153	97,92
1033	229483404	172	98,9
1034	216516303	161	96,39

Lambda

Sample ID	Total PE reads	%PE reads used	% C's in CPG methylated
1023	293899737	6061	0,16
1024	254750937	4992	0,11
1025	246833876	5618	0,21
1026	218842948	3705	1,92
1027	216451056	3692	0,79
1028	258687374	4209	3,44
1029	231908301	4299	0,27
1030	223595697	5121	0,13
1031	237301449	5338	0,16
1032	229815714	4236	0,24
1033	229483404	4225	0,22
1034	216516303	3523	0,37
